# Feasibility study of a novel wireless localization technique using radiofrequency identification markers for small and deeply located lung lesions

**DOI:** 10.1016/j.xjtc.2021.11.019

**Published:** 2022-02-19

**Authors:** Yojiro Yutaka, Toshihiko Sato, Satona Tanaka, So Miyahara, Akihiro Yoshizawa, Satoshi Morita, Hiroshi Date

**Affiliations:** a Department of Thoracic Surgery, Kyoto University Hospital, Kyoto, Japan; c Department of Diagnostic Pathology, Kyoto University Hospital, Kyoto, Japan; b Department of General Thoracic, Breast, and Pediatric Surgery, Fukuoka University Hospital, Fukuoka, Japan; d Department of Biomedical Statistics and Bioinformatics, Kyoto University Graduate School of Medicine, Kyoto, Japan

**Keywords:** wedge resection, radiofrequency identification, thoracosopy, small, deep, pulmonary nodule, 3D, 3 dimensional, CBCT, cone-beam computed tomography, CT, computed tomography, EMN, electromagnetic navigation, GGO, ground-glass opacity, NiTi, nickel titanium, RFID, radiofrequency identification

## Abstract

**Objectives:**

To evaluate the safety and efficacy of a novel wireless localization technique that uses radiofrequency identification markers for small and deep lung lesions.

**Methods:**

Preliminary use of the device was retrospectively evaluated in 2 Japanese centers. Under general anesthesia, a marker was placed as close as possible to the tumor via computed tomography-guided bronchoscopy in a hybrid operation theater. Surgeons located the marker without lung palpation using a detection probe the tone of which changed to indicate the marker-probe distance. Efficacy was defined as functional marker placement (bronchoscopy time and marker position) and deep margin distance.

**Results:**

Twelve markers were placed for 11 lesions (mean size, 6.8 ± 2.7 mm) located at a mean depth from the pleura of 11.4 ± 8.4 mm (range = 0-26.0 mm). Of 12 markers, 7 markers (58.3%) were placed within 10 mm from the lesion in 25.5 ± 14.4 minutes. For the 11 wedge resections, markers were placed at a mean distance of 6.7 mm (range, 0-13.0 mm) from the lesion and a mean distance of 14.4 mm (range, 3.0-42.0 mm) from the pleura. All markers were recovered without complications, and all tumors were resected with negative margins. For 5 lesions >10 mm deep to the pleura (mean depth, 18.9 ± 5.5 mm; range, 11.0-26.0 mm), the median depth of the surgical margin was 11.6 ± 2.1 mm (range, 9.0-14.0 mm).

**Conclusions:**

Radiofrequency identification marking was safe and precisely localized small lung lesions, including their depth.

## Graphical abstract

**Figure:**
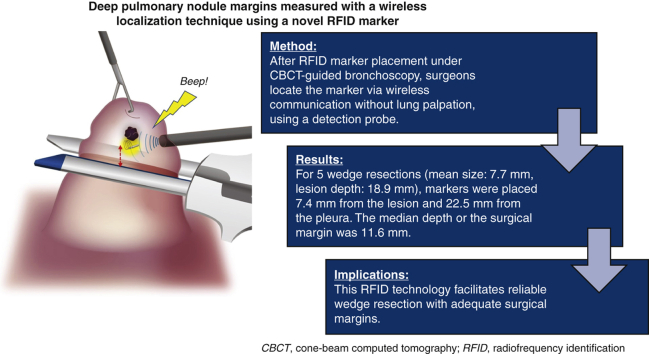

**Figure:**
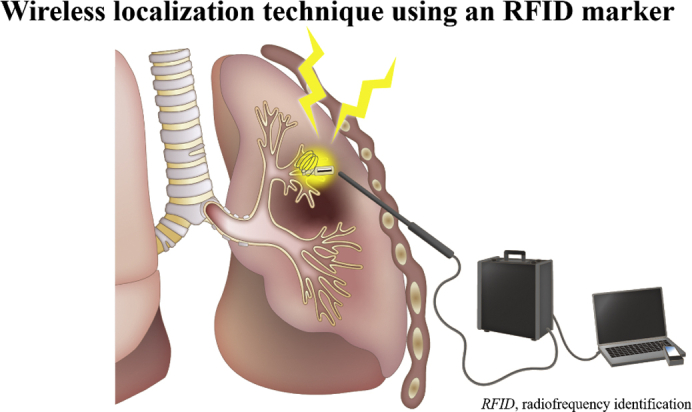
Precise wireless localization with an RFID marker.

**Central Message**

A novel localization technique using RFID wireless communication enables precise wedge resection of small and deep lung lesions.

**Perspective**

Radiofrequency identification (RFID) markers were successfully introduced, clinically, for small and deep lung lesions. The markers were fixed in the airway via bronchoscopy and provided accurate positional information, including depth. This technology facilitates reliable wedge resection with adequate surgical margins.

See Commentaries on pages 196 and 198.

Resection of small nonpalpable ground-glass opacity (GGO) lung lesions is sometimes challenging under minimally invasive thoracoscopic surgery. For subcentimeter nodules >5 mm deep to the visceral pleura, the probability of localization failure is 63%.^1^ Because no current localization techniques reliably measure surgical margin depth, localization failure rates increase with increasing depth.

We developed a novel localization technique using radiofrequency identification (RFID) technology to provide accurate positional information, including lesion depth.^2^, ^3^, ^4^ We previously reported proof of concept using a prototype in a canine experimental model. Improving the prototype enabled extending the maximum range of effective detection from 7 mm to 30 mm to adapt to the human body. RFID microchips with nickel titanium (NiTi) coil anchoring were designed to fix in the airway via bronchoscopy and can be localized with wireless communication.

We introduced this wireless localization technique in an initial human study in September 2019.^5^ Although the nodule in the first human case, a 7-mm subsolid nodule, was successfully localized by our RFID system, subpleural tumors raised an issue regarding whether a marker could provide 3-dimensional (3D) positional information, including depth. Therefore, we planned the current study to address this question. Our objective was to evaluate the safety and efficacy of this novel RFID localization technique for small, deep lung lesions during minimally invasive thoracoscopic surgery.

## Methods

We performed a preliminary study in September 2019 to assess the safety and efficacy of our wireless localization technique using an RFID marking system at Kyoto University Hospital and Fukuoka University Hospital. A retrospective chart review for the first consecutive 11 patients in wedge resections was then performed in October 2020. All patients provided written informed consent for inclusion, and the ethics committees of both hospitals approved the study protocol (safety and feasibility study of localization for small lung nodules using RFID markers: R2599; approved September 9, 2020).

### Patients

Patients with pulmonary lesions suspicious for cancer who were referred for diagnostic resection from September 2019 to October 2020 were assessed for eligibility. The inclusion criteria were any of the following conditions: pulmonary lesion expected to be invisible and poorly palpable under thoracoscopy owing to the predominant morphological feature of GGO on computed tomography (CT); lesion size <1 cm; lesion location >1 cm from the pleural surface; lesion with difficult-to-determine resection margins because of tumor location at the border of a segmental plane; expected intrapleural adhesions associated with prior lung resection or radiation therapy that would negatively influence identifying the tumor location.

### RFID Marking System

**Figure 1:**
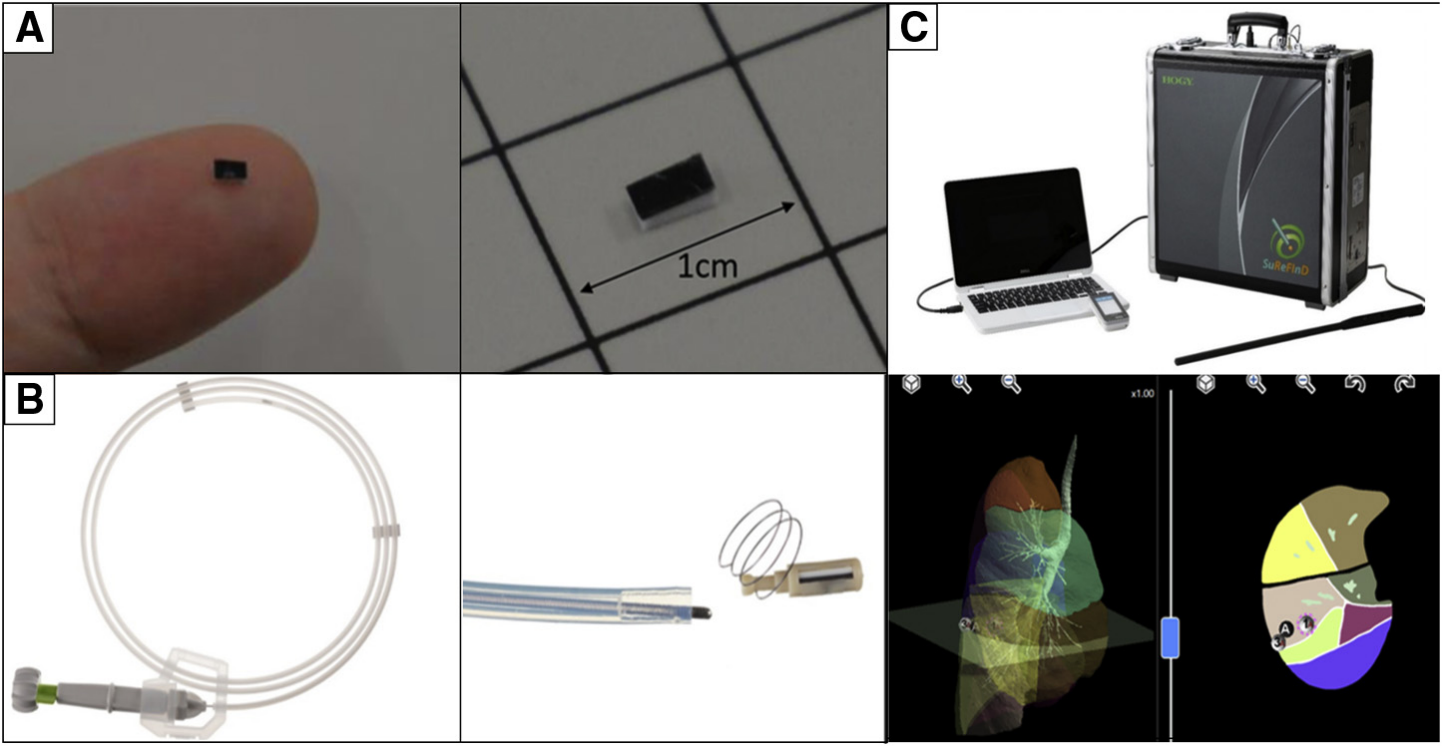
Components of the radiofrequency identification (RFID) lung marking system. A, Passive RFID tag (13.56 MHz; 3.2 × 1.6 × 0.9 mm). B, Delivery device, which is used through a bronchoscope with a 2-mm working channel. C, Detection probe (diameter, 10 mm) with a signal-processing device.

The RFID system comprises the following components: RFID marker (3.2 × 1.6 × 0.8 mm) delivery device with 5-mm NiTi coil anchors that can pass through the 2-mm working channel of a bronchoscope (Asahi Intecc) (Figure 1, *A* and *B*); wand-shaped probe (10-mm diameter, 30-mm effective range); and signal-processing unit with 3D lung imaging software on which the anatomical positions of the lesion and bronchoscopically delivered marker are registered (Welcat) (Figure 1, *C*). Briefly, the RFID marker is activated by the electromagnetic field produced by the wand-shaped probe, which acts as both a power supply and a receiver antenna.^2^, ^3^, ^4^, ^5^ The strength of the signal received by the probe is converted to 5 gradual changes in sound pitch by the signal-processing unit, with the pitch increasing as the probe approaches the marker. The RFID markers are loaded with the coil folded in the tip of the delivery device, and the coils are designed to be extended safely in the airway as a pushing wire is slowly advanced (Video 1). Each marker has a unique identification number that can be recognized. Detected marker positions are displayed in real time on a 3D lung image, where the activated marker's position flickers red.

**Video 1:**
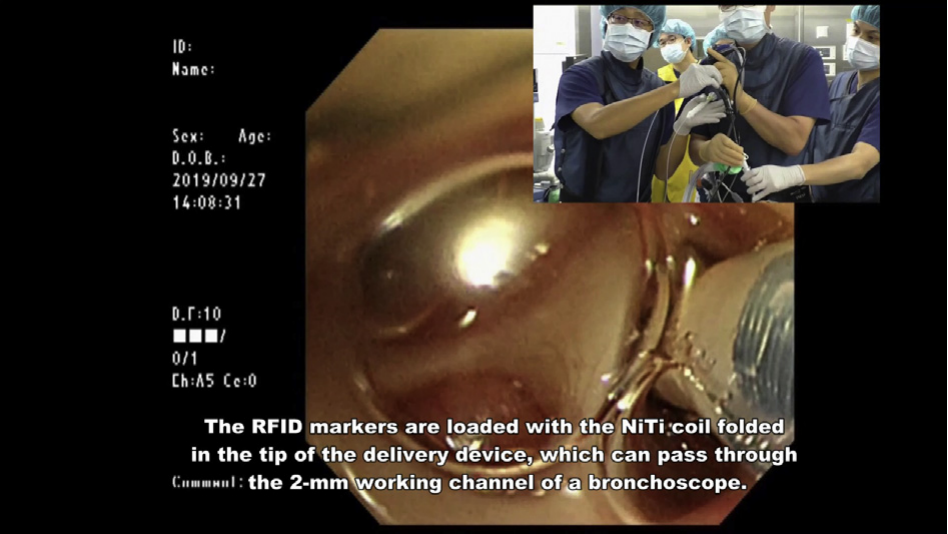
The radiofrequency identification (RFID) markers are loaded with the nickel titanium (NiTi) coil folded in the tip of the delivery device, which can pass through the 2-mm working channel of a bronchoscope. The coil is designed to be extended safely in the airway as a pushing wire is slowly moved forward while rotating the handle of the device. RFID markers with 5-mm diameter coils remain firmly fixed in the bronchi. Video available at: https://www.jtcvs.org/article/S2666-2507(22)00070-0/fulltext.

### RFID Marking Procedure

**Figure 2:**
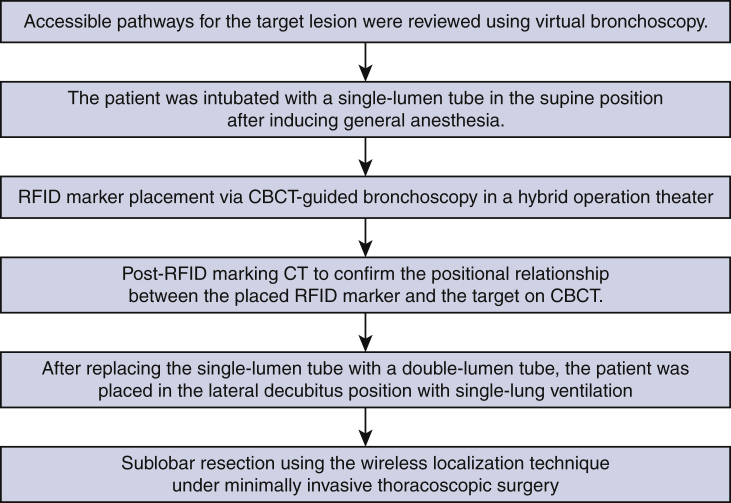
Steps in the radiofrequency identification (*RFID*) marking procedure. *CBCT*, Cone-beam computed tomography; *CT*, computed tomography.

**Figure 3:**
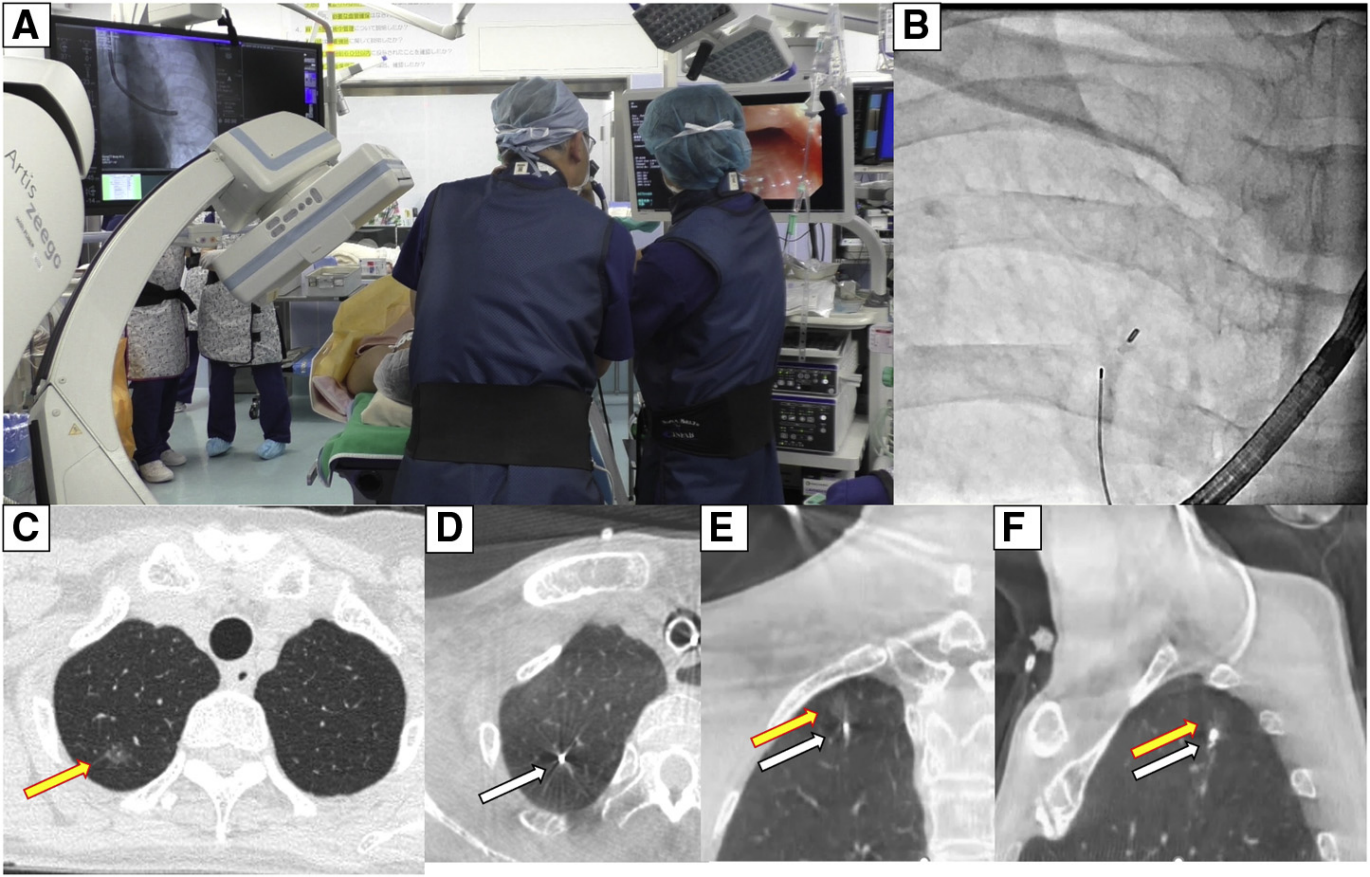
Radiofrequency identification (RFID) marking procedure using cone-beam computed tomography (CBCT) in a hybrid operation theater. A, RFID marker placement was conducted using CBCT in a hybrid operation theater just before surgery. B, Intraoperative fluoroscopy showing the RFID marker successfully placed in the airway. C, Preoperative computed tomography (CT) image showing a 5-mm lesion with pure ground-glass opacity located 15.3 mm from the pleura. D-F, Post-RFID-marking CT image showing the marker placed near the target with no apparent adverse effects (transverse, coronal, and sagittal planes, respectively).

Figure 2 shows the RFID marking procedure. Pathways for accessing the target lesion were reviewed on 3D images reconstructed by Synapse Vincent software (Fujifilm Medical) from Digital Imaging and Communications in Medicine CT data in 0.5-mm slices. Marker placement was performed in a hybrid operation theater using cone-beam CT (CBCT) just before surgery (Artis zeego; Siemens Healthcare). First, the patient was intubated with a single-lumen tube in the supine position under general anesthesia. Before marker placement, CBCT was performed during breath-holding to confirm the visibility of the target lesion. A bronchoscope (BF-P290; Olympus, Tokyo, Japan) was inserted via the intubation tube during ventilation and advanced to the target subsegmental bronchus (Figure 3, *A*). Next, an RFID delivery catheter was inserted through a working channel and advanced close to the target under CBCT guidance. After the tip of the RFID delivery catheter was confirmed to be near the target after several repeat CT inspections, the tip of the delivery catheter was finely adjusted within 10 mm of the target in the vicinity of or proximal to the target. By rotating the device's handle, the RFID marker was finally released under fluoroscopic view to fix in the peripheral airway with the expanded anchoring coil (Figure 3, *B*). A final CT scan was performed during breath-holding at a maximum airway pressure of 10 cm H_2_O after RFID marker placement to confirm the 3D positional relationship between the placed RFID marker and the target (Figure 3, *C-F*). After replacing the single-lumen tube with a double-lumen tube, the patient was placed in the lateral decubitus position for subsequent diagnostic wedge resection (Video 2).

**Video 2:**
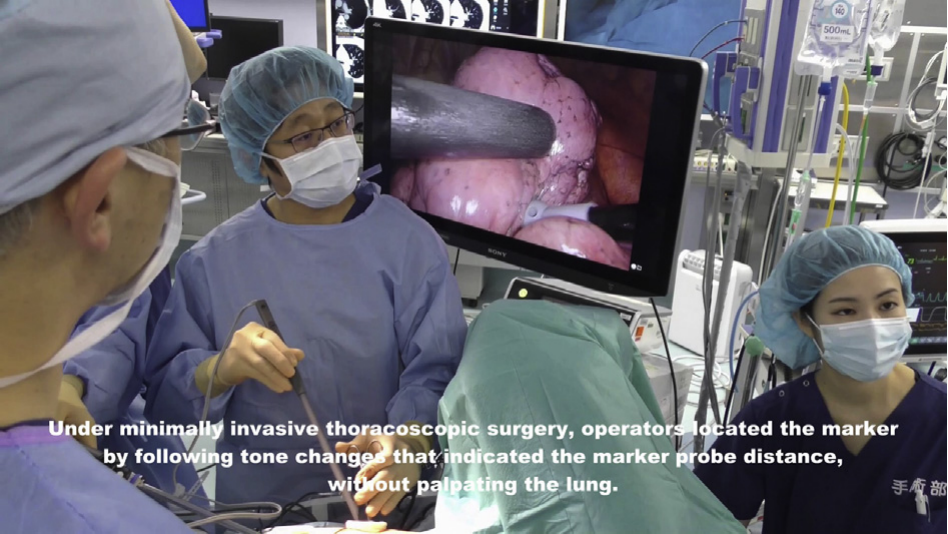
Radiofrequency identification (RFID) marker placement. Under general anesthesia, cone-beam computed tomography (CBCT)-guided bronchoscopy was performed to place a radiofrequency identification marker as close as possible to the tumor. A bronchoscope was advanced to the target subsegmental bronchus according to virtual bronchoscopic guidance. An RFID delivery catheter was inserted through a working channel and advanced close to the target under CBCT guidance. After fine adjustment of the tip of the delivery catheter proximal to the target, the RFID marker was finally released by rotating the handle of the device. The marker was checked to ensure it was fixed in the peripheral airway under fluoroscopic view, with the expanded anchoring coil. A final CBCT examination was performed to check the positional relationship between the placed RFID marker and the lesion. Under minimally invasive thoracoscopic surgery, operators located the marker by following tone changes that indicated the marker–probe distance, without palpating the lung. Video available at: https://www.jtcvs.org/article/S2666-2507(22)00070-0/fulltext.

### Wedge Resection With Wireless Marker Localization

**Figure 4:**
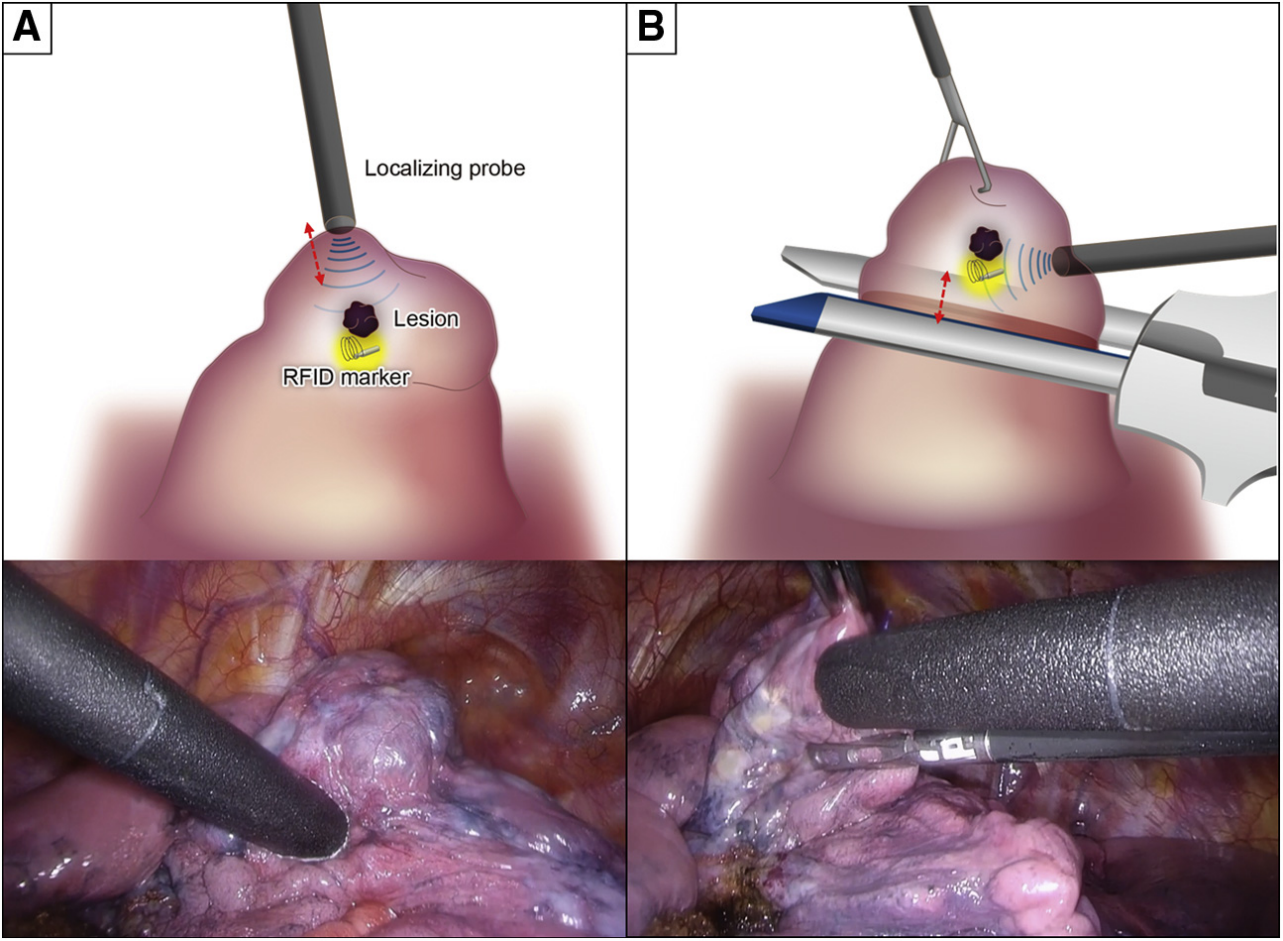
Detection method using the radiofrequency identification (*RFID*) marker to determine the deep margin resection line. A, Primary detection to identify the nearest pleural point from the marker. B, Secondary detection from differential directions to identify the marker depth from the pleura in the elevated lung.

Tumors were excised by wedge resection without lung palpation via 3-port video-assisted thoracoscopic surgery with a 15-mm incision where wound retractors (XXS size) (Alexis Wound Retractor; Applied Medical) were placed as follows: seventh intercostal space at the midaxillary line, fourth intercostal space at the anterior axillary line, and in the triangle of auscultation. After placing the 3 ports, the lobe of interest was scanned with the localizing probe. Because the probe has directivity in wireless communication, the angle of incidence between the probe and the pleural surface was approached closer to 90° during marker exploration by the probe. The RFID markers were localized through the nearest port from the lesion. Operators located the marker by following tone changes corresponding to the marker–probe distance. The time from beginning the detection to recognizing the marking site upon hearing the highest-pitched sound was recorded. This first detection revealed the nearest pleural point from the marker, and the point was marked with a 4–0 polydioxanone suture or electrocautery (Figure 4, *A*). After elevating the sutured pleural point with a thoracoscopic grasper, the marker was carefully scanned with the localizing probe from the sides to locate the marker (Figure 4, *B*). This secondary detection revealed the depth of the RFID marker more clearly in conjunction with the first detection. The expected resection line was adjusted by rescanning with the probe, and the target lesion was removed with the marker using a linear stapler or electrocautery, with a planned deep resection margin of 10 mm (the distance to the tip of the probe). We also confirmed that the marker had not been left behind in the remaining lung. After wedge resection, we examined the positional relationship between the lesion and the RFID marker (Video 3). All procedures, from marker placement to surgery, were performed by either Y.Y. or T.S., both of whom had sufficient experience using this system.

**Video 3:**
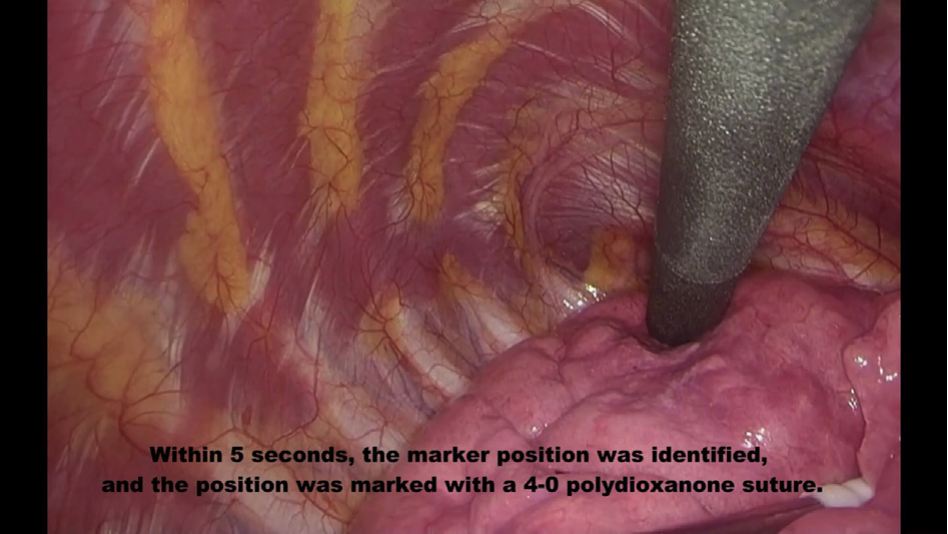
Wedge resection. Radiofrequency identification (RFID) marker was placed 3 mm from the lesion. Within 5 seconds, the marker position was identified, and the position was marked with a 4-0 polydioxanone suture. To determine the depth of the deep margin, the lung was retracted upward, and the marker's location was carefully checked. After resecting the lung tissue by wedge resection, we examined the positional relationship between the lesion and the RFID marker. Video available at: https://www.jtcvs.org/article/S2666-2507(22)00070-0/fulltext.

### Safety and Efficacy

**Figure 5:**
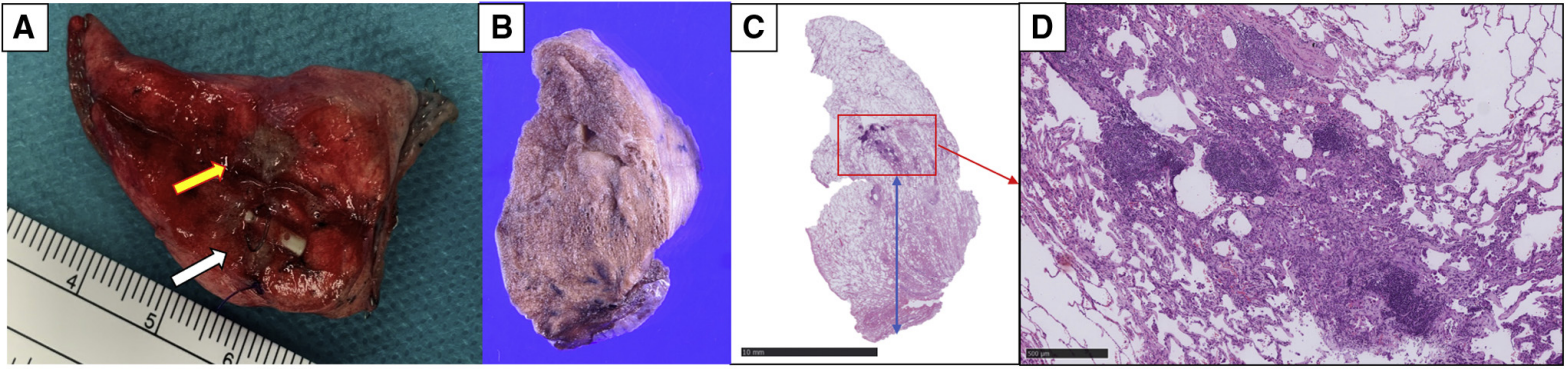
Macroscopic findings and pathological examinations of lung tissue excised by wedge resection, confirming the positional relationships between the radiofrequency identification (RFID) marker and the target. A, Macroscopic findings. This lesion was finally diagnosed as a 5-mm minimally invasive adenocarcinoma, and it was removed with a deep margin of 12 mm (*yellow arrow* indicates tumor, *white arrow* indicates RFID marker). B-D, Microscopic findings showing that the lesion was a 5-mm minimally invasive adenocarcinoma with a 1-mm invasive component excised with a deep margin of 12 mm (*red square* indicates tumor, *blue bar* indicates deep margin).

The safety of RFID marking was evaluated according to CT findings immediately after marker placement and according to intraoperative marker dislodgement, indicated by bronchoscopy procedure time and marker position (distance to the lesion and depth from the pleura). Efficacy was evaluated according to functional marker placement and tumor localization status, suggested by the tumor recovery rate and marker localization time. Deep margin status was evaluated intraoperatively after removing the staplers (Figure 5, *A*). To minimize variation during margin measurement, this procedure had been matched in the 2 institutions before beginning the current study (Figure 5, *B-D*).

### Statistical Analysis

Numerical variables were presented as mean ± SD. Categorical variables were summarized in a frequency distribution table. Descriptive statistics were performed to describe the main features of numerical and categorical data, with simple summaries.

## Results

### Patient Characteristics

Twelve RFID markings were performed for 11 lesions in 11 patients (Table 1). The median patient age was 65.0 years (range, 42.0-78.0 years). The mean pulmonary lesion measured 6.8 ± 2.7 mm (range, 3.0-11.0 mm), and lesions were located at a mean depth from the pleura of 11.4 ± 8.4 mm (range, 0-26.0 mm). The mean consolidation to tumor ratio was 63.2 ± 45.8 (range, 0-100). Four (36.4%) lesions were located in the right upper lobe, 1 (9.1%) in the right lower lobe, 3 (27.3%) in the left upper lobe, and 3 (27.3%) in the left lower lobe. All operations (n = 11) were accomplished as wedge resections.

**Table 1:** Patient and lesion characteristics (n = 11)

Variable	Result	Range
Age (y)	62.5 ± 11.4	42-78
Male sex	7 (63.6)	
Tumor size (mm)	6.8 ± 2.7	3.0-11.0
Depth from the pleura (mm)	11.4 ± 8.4	0-26.0
Consolidation to tumor ratio	63.2 ± 45.8	0-100
Patients who previously underwent an intrathoracic operation	2 (18.2)	
Lesion location		
Right upper lobe	4 (36.4)	
Right middle lobe	0	
Right lower lobe	1 (9.1)	
Left upper lobe	3 (27.3)	
Left lower lobe	3 (27.3)	

Values are presented as n (%) or mean ± SD.

### Safety

#### CT after RFID marker placement

**Figure 6:**
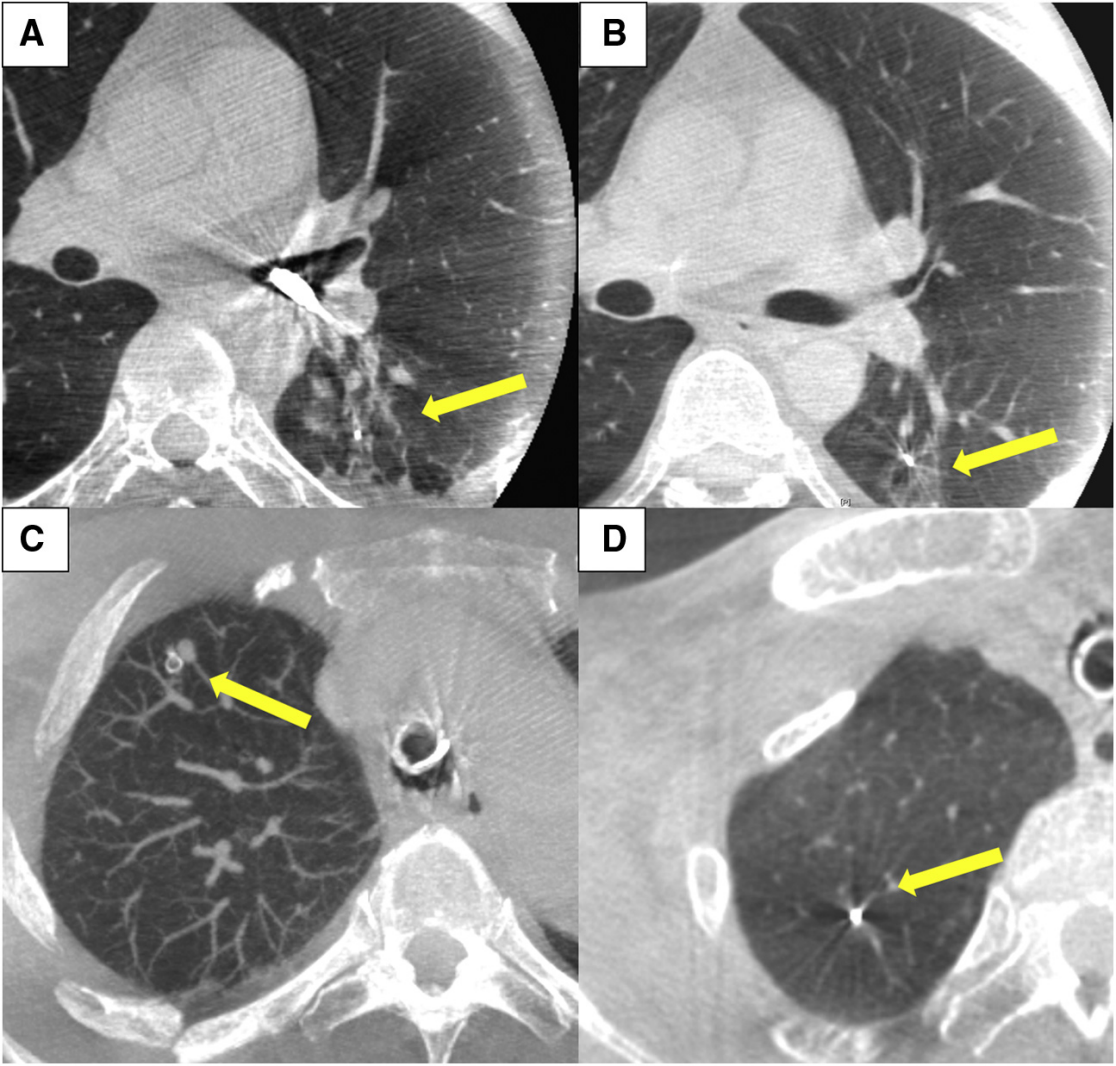
Representative computed tomography (CT) findings of the radiofrequency identification (RFID) marking procedure. A, CT findings of possible intrapulmonary injury associated with device manipulation before releasing the RFID marker. B, CT after marking revealed that the intrapulmonary shadow was less obvious, and there was no apparent intrapulmonary hemorrhage. C, CT after RFID marking revealed that the marker was fixed by a nitinol (NiTi) coil anchor. D, There was no apparent intrapulmonary injury after RFID marking.

Bronchoscopy after marker placement showed no intrabronchial bleeding. All pulmonary lesions and bronchoscopically delivered RFID markers were confirmed on CBCT. Procedural CT revealed no apparent adverse effects, including pneumothorax and intrapulmonary hemorrhage associated with marker injection from the device (Figure 3, *C-F*). During bronchoscopy in 1 patient, seeking procedures to reach the target might have injured the pulmonary parenchyma; linear opacities along the bronchus with some local atelectasis were identified before releasing the RFID marker. However, after releasing the marker, these shadows were not exacerbated and were obscure on the final CT after lung recruitment (Figure 6, *A* and *B*). Representative CT findings are shown in Figure 6, *C* and *D*.

#### Intrapulmonary marker fixation

None of the delivered RFID markers could be observed bronchoscopically. The maximum bronchial diameter where the RFID markers were installed was 1.8 mm. Regarding intrapulmonary fixation of the markers, although intraoperative manipulations with strong traction were required in 2 patients with severe intrapulmonary adhesions, no intraoperative dislodgement occurred.

### Efficacy

#### Functional marker placement

The mean duration of the marking procedure was 25.5 ± 14.4 minutes (range, 13-54 minutes). RFID markers were placed at a mean distance of 6.7 ± 5.2 (range, 0-13.0 mm) from the lesion and a mean distance of 14.4 ± 10.2 mm (range, 3-42.0 mm) from the pleura. In wedge resection, 58.3% (7 out of 12) of the markers were successfully placed within 10 mm from the target (Table 2).

**Table 2:** Outcomes of 12 radiofrequency identification (RFID) markings for wedge resection of 11 lesions

Variable	Mean ± SD	Range
Marking procedure time (min)	25.5 ± 14.4	13.0-54.0
No. of CT scans	4.3 ± 0.8	3.0-5.0
Marker location on CT		
Distance to lesion (mm)	6.7 ± 5.2	0-13.0
Distance from pleura (mm)	14.4 ± 10.2	3.0-42.0
Marker localization time (sec)	4.3 ± 1.5	2.0-8.0
Tumor recovery rate (%)	100	
Distance from the marker to the lesion (mm)	3.8 ± 3.3	2.0-8.0
Depth of the surgical margin (mm)	11.8 ± 2.0	9.0-15.0

*CT*, Computed tomography.

#### Tumor localization status

No pleural change, including indentation and color change, was identified thoracoscopically. All tumors were detected by the probe with wireless localization. The marker was localized in 4.3 ± 1.5 seconds (range, 2.0-8.0 seconds). All tumors were removed along with the placed markers. All surgical margins were negative, and the mean depth of the surgical margin evaluated pathologically was 10.8 ± 0.6 mm (range, 8.0-13.0 mm).

### Wedge Resection Cases

The outcomes of the 11 wedge resections are summarized in Table 3. The mean lesion size was 6.8 ± 2.7 mm (range, 3.0-11.0 mm), and the mean distance from the pleura was 11.4 ± 8.2 mm (range, 0-26.0 mm), Twelve RFID markers were placed for subsequent wedge resection. Two markers were required in 1 patient because of limitations associated with the bronchial anatomy (patient 8). In the 11 wedge resections, markers were placed at a mean distance of 6.7 ± 5.2 mm (range, 0-13.0 mm) from the lesion and at a mean distance of 14.4 ± 10.2 mm (range, 3.0-42.0 mm) from the pleura. The mean wedge resection time (excluding chest opening and closure) in 9 patients without intrathoracic adhesion, was 28.8 ± 10.4 minutes (range, 16-46 minutes). Tumors were macroscopically resected with a mean deep margin of 11.8 ± 2.0 mm (range, 9.0-15.0 mm). Pathological examination revealed 4 adenocarcinomas, 2 minimally invasive adenocarcinomas, 1 adenocarcinoma in situ, 3 metastatic tumors, and 1 benign tumor. One wedge resection (patient 11) was converted to lobectomy because intraoperative pathological findings revealed a 10-mm invasive adenocarcinoma with a solid component.

**Table 3:** Details of the 11 wedge resections using 12 radiofrequency identification (RFID) markers

Patient	Lesion	Size on CT (mm)	C/T ratio	Depth from the pleura (mm)	Distance to the lesion (mm)	Marker depth (mm)	Pathology	Macroscopic margin (mm)	Pathological margin (mm)	p-TNM
1	Rt S10	6.0	100	2	5	7	AD	15	13	T1a N0 M0
2	Lt S6	5.0	0	7	11	14	MIA	12	10	T1mi N0 M0
3	Lt S6	11.0	100	11	4.3	14.8	AD	13	12	T1b N0 M0
4	Rt S3	4.0	100	9	0	8	Meta	10	10	–
5	Rt S1	3.0	100	20	8	16.5	Meta	14	12	–
6	Lt S1+2	6.0	100	17	0	17	Granuloma	9	10	–
7	Lt S1+2	10.0	62	0	11	3	AD	11	10	T1a N0 M0
8	Lt S3	8.2	0	20.6	12	22	AIS	10	8	Tis N0 M0
					13	11				
9	Lt S1+2	5.1	0	9.1	0	10	MIA	14	12	T1mi N0 M0
10	Lt S10	6.0	33.3	4	3	7	Meta	10	11	–
11∗	Rt S3	10.1	100	26	12.5	42	AD	12	11	T1b N0 M0
Mean ± SD		6.8 ± 2.7	63.2 ± 45.8	11.4 ± 8.4	6.7 ± 5.2	14.4 ± 10.2	–	11.8 ± 2.0	10.8 ± 0.6	–

*CT*, Computed tomography; *C/T* ratio, consolidation/tumor ratio; *p-TNM*, pathological tumor-node-metastasis; *Rt*, right; *S*, segment; *AD*, adenoma; *Lt*, left; *MIA*, minimally invasive adenocarcinoma; *Meta*, metastatic; *AIS*, adenocarcinoma in situ.

∗ Patient 11 was converted from wedge resection to lobectomy due to unanticipated aggressive pathological findings.

**Figure 7:**
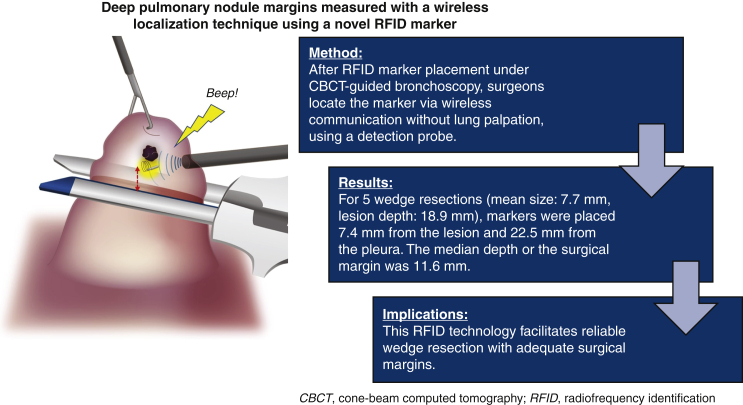
Outline of the wireless localization technique using radiofrequency identification (*RFID*) markers. The deep surgical margin could be measured using a novel RFID marker and wireless communication. *CBCT*, Cone-beam computed tomography.

For 5 lesions >10 mm deep to the pleura (mean depth, 18.9 ± 5.5 mm; range, 11.0-26.0 mm), markers were placed at a mean distance of 7.4 ± 5.3 mm (range, 0-12.5 mm) from the lesion and at a mean distance of 22.5 ± 11.2 mm (range, 14.8-42.0 mm) from the pleura. The mean depth of the surgical margin was 11.6 ± 2.1 mm (range, 9.0-14.0 mm) (Figure 7). All patients were recurrence-free as of September 11, 2021.

## Discussion

RFID is a wireless communication technology that can transfer data or track objects using radio waves. The technology was originally developed for military radar systems in the 1940s and is used worldwide for patient identification, and for tracking pharmaceutical products or surgical instruments.^6^, ^7^, ^8^ Technical innovations in downsizing RFID chips has accelerated the spread of this technology, which received approval for implantation in humans from the US Food and Drug Administration in 2004. In this study, we demonstrated the safety of airway delivery of RFID markers, and the feasibility of using these markers to determine the deep surgical margins of small lung lesions during thoracoscopic surgery. Although optimal surgical margins remain undefined, because a margin to tumor size ratio <1 predicts positive margin cytology findings, which reflects local recurrence, subcentimeter pulmonary lesions should be removed with a 1-cm deep margin.^9^

Early diagnosis increases the chances of curability in cancer treatment. Accordingly, preoperative marking methods have been developed in minimally invasive thoracoscopic surgery to minimize the time from incidental identification to definitive diagnosis of small lung lesions morphologically suspicious for lung cancer. Marking methods are classified as CT-guided transthoracic marking and bronchoscopy-guided transairway marking. Transthoracic access is an option for lesions inaccessible via the bronchi; however, this approach is associated with higher pneumothorax rates (8%-50%)^10^^,^^11^ resulting from puncturing the visceral pleura, and the method is unsuitable for patients with multiple lesions. Transthoracic marking carries other risks, namely pulmonary hemorrhage (12%-35%) and fatal air embolism (0.02%) caused by perforating the pulmonary vein.^10^, ^11^, ^12^ Therefore, we select safer preoperative marking methods using bronchoscopy to avoid fatal complications in patients awaiting curative surgical resection.

Tumor visibility and palpability depends on the conditions of the underlying lung. Specifically, pleural adhesion can impair tumor localization, which is affected by pleural thickening or pleural detachment. To overcome this problem, we included expected intrapleural adhesion cases in this study, and we used subpleural cases to confirm the usefulness of the device and the pushability of the RFID marker. From our early experience using this RFID delivery device, we confirmed that the device could be smoothly advanced to the subpleural area, and the marker was safely released without causing pneumothorax. A firmly fixed RFID marker by the NiTi coil anchor contributed to tumor localization even in adhesion cases.

A recent multi-institutional prospective trial revealed that the successful resection rate using dying methods was 87.7%.^13^ The most significant factor affecting resection failure was lesion depth, and the resection failure rate increased as tumor depth from the pleura increased. Because of the limited dyed area to the pleural surface, deep resection lines cannot be easily determined using only dying methods. Microcoil localization with intraoperative fluoroscopy overcomes this issue. Finley and colleagues^14^ reported a 93% (27 out of 29) successful resection rate for 12.7-mm lesions located 15.5 mm from the pleura by wedge resection using this technique, although localization failure owing to coil migration occurred in 3% of the patients.^14^ In that study, 2 coils were placed deep to the nodule and on the visceral pleural surface under local anesthesia by an interventional chest radiologist for subsequent visualization by intraoperative fluoroscopy and video-assisted thoracoscopic surgery. In contrast, in the current study, wedge resection using a single RFID marker conveniently enabled determining the deep margin resection line after confirming the RFID marker position. We have not experienced dislocation of RFID markers; however, markers were installed only in bronchi measuring <1.8 mm. Removing migrated markers is recommended, considering the potential harmful effects associated with residual markers; therefore, confirming the bronchial diameter before marker placement is advised. To minimize serious complications associated with marker migration, serial procedures from marking to surgery are ideally performed in a hybrid operation room equipped with CBCT, which helps reduce medical staff and patient stress compared with performing these procedures separately.^15^

To take full advantage of our RFID marking system, markers must be delivered through a bronchoscope in the vicinity of the tumor. Although intrapulmonary sites accessible via bronchoscopy depend on an individual's bronchial anatomy, this critical issue may be overcome using CBCT-guided bronchoscopy. A recent study revealed that adding electromagnetic navigation (EMN) to CBCT significantly increased bronchoscopic navigation success to 87.5% compared with CBCT or EMN alone (76.3% and 52.2%, respectively).^16^ Because EMN can potentially guide the bronchoscope to an ideal site adjacent to targets that appear unreachable by conventional bronchoscopy,^17^^,^^18^ EMN under CBCT imaging might improve marker placement accuracy in less time and reduce the radiation exposure.

There are limitations in this study affecting the generalizability of the results. First, the lack of a priori sample size calculations and safety thresholds is a major limitation. This was a retrospective review of very early clinical experience using RFID markers. To justify the results obtained in this study, methodological refinements are required in a prospective study. The second limitation is that all procedures were performed by 2 surgeons familiar with the function and characteristics of this system. Sufficient experience and understanding of this system may be prerequisites for clinical use. Because the probe has directivity in wireless communication, and detection from different directions is required to measure the RFID marker depth, the skin incision for the localization probe must be carefully planned according to the lesion position. Third, the possible effects of the RFID marker placement on the pathological findings must be evaluated in additional cases. In the current study, although no apparent artifacts interfering with the pathological interpretation of the target lesion occurred, future examination of the pathological effect of this technique is required. Finally, this new RFID marking technology also requires cost–benefit analysis to determine the best management of small lung nodules.

## Conclusions

The wireless localization technique used in this study was safe and achieved successful resection for small and deep lung lesions.

### Webcast

You can watch a Webcast of this AATS meeting presentation by going to: https://aats.blob.core.windows.net/media/Publications/AM21_TH08%20-%20Lung%20Cancer%202.mp4-PresentationPlusDiscussion-hi.mp4.

**Figure:**
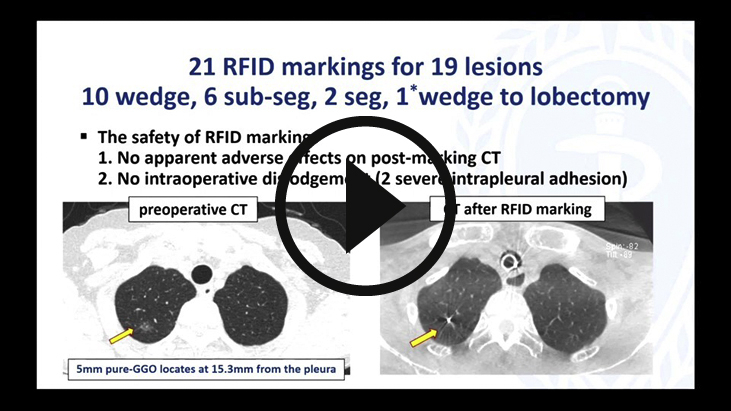

### Conflict of Interest Statement

Drs Yutaka, Sato, and Date have a financial relationship with Hogy Medical Co Ltd, which developed the radiofrequency identification system used in this study. All other authors reported no conflicts of interest.

The *Journal* policy requires editors and reviewers to disclose conflicts of interest and to decline handling or reviewing manuscripts for which they may have a conflict of interest. The editors and reviewers of this article have no conflicts of interest.

## Supplementary Data

Video 1 The radiofrequency identification (RFID) markers are loaded with the nickel titanium (NiTi) coil folded in the tip of the delivery device, which can pass through the 2-mm working channel of a bronchoscope. The coil is designed to be extended safely in the airway as a pushing wire is slowly moved forward while rotating the handle of the device. RFID markers with 5-mm diameter coils remain firmly fixed in the bronchi. Video available at: https://www.jtcvs.org/article/S2666-2507(22)00070-0/fulltext.

Video 2 Radiofrequency identification (RFID) marker placement. Under general anesthesia, cone-beam computed tomography (CBCT)-guided bronchoscopy was performed to place a radiofrequency identification marker as close as possible to the tumor. A bronchoscope was advanced to the target subsegmental bronchus according to virtual bronchoscopic guidance. An RFID delivery catheter was inserted through a working channel and advanced close to the target under CBCT guidance. After fine adjustment of the tip of the delivery catheter proximal to the target, the RFID marker was finally released by rotating the handle of the device. The marker was checked to ensure it was fixed in the peripheral airway under fluoroscopic view, with the expanded anchoring coil. A final CBCT examination was performed to check the positional relationship between the placed RFID marker and the lesion. Under minimally invasive thoracoscopic surgery, operators located the marker by following tone changes that indicated the marker–probe distance, without palpating the lung. Video available at: https://www.jtcvs.org/article/S2666-2507(22)00070-0/fulltext.

Video 3 Wedge resection. Radiofrequency identification (RFID) marker was placed 3 mm from the lesion. Within 5 seconds, the marker position was identified, and the position was marked with a 4-0 polydioxanone suture. To determine the depth of the deep margin, the lung was retracted upward, and the marker's location was carefully checked. After resecting the lung tissue by wedge resection, we examined the positional relationship between the lesion and the RFID marker. Video available at: https://www.jtcvs.org/article/S2666-2507(22)00070-0/fulltext.
